# Channeling the Natural Properties of Sindbis Alphavirus for Targeted Tumor Therapy

**DOI:** 10.3390/ijms241914948

**Published:** 2023-10-06

**Authors:** Christine Pampeno, Alicia Hurtado, Silvana Opp, Daniel Meruelo

**Affiliations:** Department of Pathology, NYU Grossman School of Medicine, New York University, New York, NY 10016, USA

**Keywords:** sindbis virus, alphavirus, cancer therapy, immunotherapy

## Abstract

Sindbis alphavirus vectors offer a promising platform for cancer therapy, serving as valuable models for alphavirus-based treatment. This review emphasizes key studies that support the targeted delivery of Sindbis vectors to tumor cells, highlighting their effectiveness in expressing tumor-associated antigens and immunomodulating proteins. Among the various alphavirus vectors developed for cancer therapy, Sindbis-vector-based imaging studies have been particularly extensive. Imaging modalities that enable the in vivo localization of Sindbis vectors within lymph nodes and tumors are discussed. The correlation between laminin receptor expression, tumorigenesis, and Sindbis virus infection is examined. Additionally, we present alternative entry receptors for Sindbis and related alphaviruses, such as Semliki Forest virus and Venezuelan equine encephalitis virus. The review also discusses cancer treatments that are based on the alphavirus vector expression of anti-tumor agents, including tumor-associated antigens, cytokines, checkpoint inhibitors, and costimulatory immune molecules.

## 1. Introduction

A Sindbis viral vector platform was developed for the treatment of multiple types of tumors [1,2,3,4,5,6]. Sindbis virus (SV) is an enveloped, single-stranded, positive-sense RNA virus [7]. Like other members of the *Alphavirus* genus and *Togavirus* family, SV is transmitted to vertebrates via arthropods and is hence known as an arthropod-borne or arbovirus [7]. Alphaviruses are classified as either Old World or New World viruses based on (1) their geographic location and relative distributions; (2) their pathogenicity in humans and animals; (3) their utilization of different host-cell proteins for replication [7,8].

SV is an Old World virus which was first isolated from mosquitos within the Egyptian Sindbis district [9]. Old World alphaviruses, such as SV, Chikungunya virus (CHIKV), Semliki Forest Virus (SFV), and Ross River virus (RRV), cause fever and arthralgia-like disease, while New World viruses like Venezuelan Equine Encephalitis Virus (VEEV), Eastern Equine Encephalitis Virus (EEEV), and Western Equine Encephalitis Virus (WEEV) cause more severe encephalitic infections.

Comprehensive reviews of alphavirus structure, expression, replication, and evolution have been published [7,10,11]. This review, however, focuses on SV and related studies that promote alphavirus vectors as effective therapies for cancer treatment.

### 1.1. Alphavirus Vectors

SV has long been recognized as an effective vector system [12,13]. Alphaviruses, in general, have many attributes that render them advantageous as gene expression vectors, including: (1) a broad host range for entry into mammalian cells [13]; (2) the formation of a replication complex in the cytoplasm of infected cells [14,15,16] that produces approximately 10^6^ copies of viral RNA which, coupled with a strong subgenomic promoter, enables very high expression levels of recombinant proteins [12,17]; and (3) RNA replication without DNA intermediates, avoiding the risk of chromosomal integration or insertional mutagenesis [18]. Finally (4), alphavirus vectors are easy to manipulate and can accommodate at least 8000 bp’s of heterologous mRNA [19].

### 1.2. Alphavirus Vectors as an Oncolytic-Virus-Mediated Therapy

Oncolytic viruses can infect and specifically replicate in tumor cells, eliciting cell death and stimulating an anti-tumor immune response. Oncolytic viruses can be DNA-based (e.g., adenoviruses, vaccinia, and herpes simplex) or RNA-based, including single- stranded, negative-sense viruses (e.g., measles, Newcastle disease, and vesicular somatitus viruses), positive-sense viruses (e.g., polio, Coxsackie, Sindbis, Semliki forest, and Venezuelan equine encephalitis viruses), or double-stranded reoviruses. As of 2021, there are 153 DNA and 70 RNA oncolytic-virus-mediated clinical trials in progress investigating the viruses as either monotherapies or in combination with other agents [20]. Recent comprehensive reviews discussed the different modalities and progression of oncolytic-virus-mediated therapies [20,21,22,23,24,25,26,27].

With Sindbis virus as a prototype, this review promotes the utilization of single-stranded RNA alphaviruses, which have thus far been under-represented, for clinical treatments of cancer. Several characteristics make alphaviruses particularly suitable as cancer-therapeutic agents: (1) like other oncolytic viruses, alphaviruses are cytopathic, causing infected cells to die via apoptosis [5,28,29]; apoptotic bodies could induce immune responses via the cross-priming of tumor-associated antigens [30,31,32]; (2) double-stranded RNA replication intermediate elicits “danger signals”, activating antiviral pathways that stimulate innate immune responses, further enhancing adaptive immunity [33,34,35,36,37,38,39]; (3) alphaviruses are known to target lymph nodes [3,31,40] and infect monocytes, macrophages, and dendritic cells [41,42]; () (4) transmitted via insect bites, alphaviruses are blood-borne, allowing recombinant vectors to be stably delivered systemically [7]; (5) the repeated administration of vectors remains efficacious [43,44,45].

## 2. Development of Sindbis Virus as an Effective Vector for Cancer Treatment

### 2.1. Vector Safety

**Figure 1:**
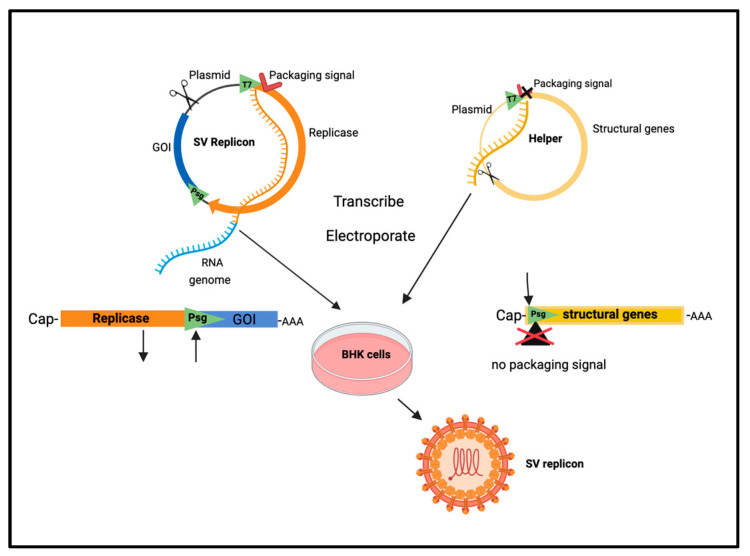
Preparation of SV vector. Plasmid and linear maps of replicon with gene of interest and helper with structural genes. T7, in vitro transcription promoter; Psg , Sindbis subgenomic promoter; GOI, gene of interest; AAA, polyA sequence. Created with BioRender.com.

Sindbis virus has the safest profile among alphaviruses. Mostly asymptomatic infections occur. When rare symptomology occurs, it predominantly involves a mild fever, rash, and arthralgia that promptly resolve [46,47,48]. To further minimize infection accompanied by symptomology, SV vectors were constructed by splitting the SV genome into two plasmid segments [12,17]. A replicon plasmid encodes the non-structural replicase proteins and contains a strong subgenomic promoter to express heterologous “genes of interest”. The viral packaging signal is removed from the separate helper DNA plasmid that encodes the structural capsid and envelope proteins that would normally be expressed from the genome subpromoter. The removal of the packaging signal prevents the inclusion of the helper RNA strand into the virus particle, precluding subsequent virus formation due to the absence of the necessary structural proteins. The SV sequences, preceded by a bacteriophage promoter, are transcribed in vitro, and RNA is transfected into baby hamster kidney cells (BHKs) to produce SV particles that only contain a replicon genome and are propagation-defective vectors (see Figure 1).

### 2.2. Early Investigations: Engineered Targeting

Initial studies to harness SV for cancer treatment presumed the need to design vectors that target tumor cells while failing to infect normal cells. The discovery by Dubuisson et al. [49] of a mutated site within the SV envelope E2 protein that impeded viral entry, but not virus particle assembly and release, facilitated the creation of a chimeric SV E2 vector that could target cells. Two IgG binding domains of the Staphylococcus aureus protein A were inserted between amino acids 71 and 74 of the E2 protein [50]. Protein A, having a strong affinity for the Fc region of many mammalian IgG antibodies [51], allowed the modified vector to target many types of both adherent and suspension tumor cells only when coupled with monoclonal antibodies that react with cell-surface tumor antigens [50].

A more specific chimeric envelope vector was created via the insertion of human chorionic gonadotrophic glycoprotein α and β subunits within the same E2 site. This vector was able to infect human choriocarcinoma cell lines that express luteinizing hormone and chorionic gonadotropin receptors in a dose-dependent manner [52].

When the modified envelope vectors were applied to treat in vivo tumors, unexpectantly, the unmodified control SV vectors were as effective at decreasing the tumor burden without infecting normal cells [5,6,53,54,55]. These surprising results led to the examination of SV vector localization in mouse tumor models.

### 2.3. In Vivo Imaging Studies

Unmodified SV vectors that expressed LacZ were initially used for the in vivo localization of an injected vector [54]. C.B-17-SCID (severe combined immunodeficiency) mice were injected subcutaneously with baby hamster kidney (BHK) or human tumor cell lines representing colon, ovarian, pancreatic, and hepatocellular malignancies. The SV-LacZ vector was injected intraperitoneally (i.p.) after tumors were well established. Tumors in mice that received the SV-LacZ vector began to decrease in size by day 6 to 7 after treatment while the control mice receiving physiological saline required euthanasia by day 12 due to high tumor burdens. After 30 days of treatment, complete tumor regression was observed in four of the five mice treated with the SV-LacZ vector. The immunohistochemistry of the treated mice showed LacZ staining in the necrotic areas of the tumors but not in viable tissue. Serial tumor sections stained with antibodies to both LacZ and an endothelial marker, Factor VIII, suggested a colocalization of the SV vector with the tumor vasculature. Later imaging studies which used two different near-infrared fluorescent probes, Qtracker and AngioSense, for the in vivo molecular imaging of tumor vasculature provided bio-optical imaging evidence that SV vectors correlate with vascular leakiness in tumors [56]. The association of tumor vascularization, vascular leakiness, and tumoral virus distribution was confirmed in mouse studies, using positron emission tomography (PET) with radioactive thymidine kinase substrates to image the SV-thymidine kinase vector infection of tumors [57]. Their evolution from arboviruses provides SV vectors the advantage of stable delivery through the bloodstream to target tumors and metastases.

The advent of bioluminescent in vivo imaging systems (IVISs) enabled the noninvasive monitoring of SV vector delivery and its infection and eradication of tumor cells in live mice. Human ES-2 cells, derived from a clear cell ovarian carcinoma known for its resistance to many chemotherapeutic agents [58], were injected intraperitoneally (i.p.) into immunodeficient C.B-17-SCID mice and 5 days later, an SV vector expressing firefly luciferase (SV-Fluc) was i.p. injected. Bioluminescence measurements showed the specific tumor infection with the Sindbis/Fluc vector throughout the peritoneal cavities of the ES-2–inoculated mice. Control tumor-free mice showed low signals in fat deposits which disappeared after a second vector injection.

**Figure 2:**
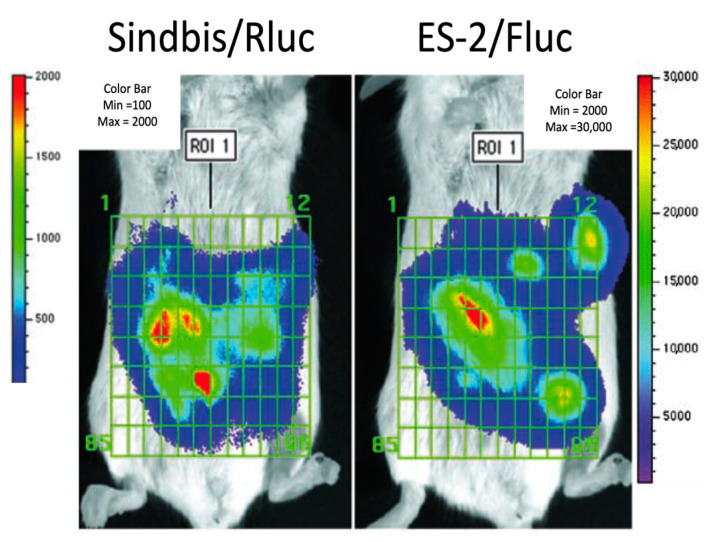
Sindbis/Rluc infection colocalized with the metastasized ES-2/Fluc tumors in the peritoneal cavity as determined by the IVIS system. Color bars show photo counts [5].

To confirm the degree and specificity of the Sindbis infection of the tumor cells, imaging studies were conducted that measured independent bioluminescent signals from ES-2 tumor cells and SV vector [5]. The cell lines were transfected with firefly luciferase expression plasmids (Fluc), while the SV vector expressed Renilla luciferase (Rluc). Firefly luciferase uses D-luciferin, whereas Renilla luciferase uses coelenterazine to generate bioluminescence. Both luciferases are highly substrate-specific and do not cross-react, however, coelenterazine has a shorter half-life [59]. For a quantitative analysis, bioluminescence signals were generated in the same animal by first injecting the short-lived Renilla substrate; after signal fading, this was followed by D-luciferin. Measurements were acquired and quantitated using Living Image software (Xenogen). A significant correlation (*p* < 0.0001) was established between the signals from SV-Rluc and Fluc tumor cells, indicating that a single i.p. delivery of Sindbis vectors led to the efficient infection of the metastasized ES-2 tumor cells throughout the peritoneal cavity (Figure 2). Further imaging experiments also showed that Sindbis vectors target syngeneic [5] and spontaneous [6] tumors in immune-competent mice. Therefore, SV vectors infected human and mouse cell-line-derived tumors as well as in vivo-generated mouse tumors.

### 2.4. Sindbis Virus Infection, Tumor Targeting, and the 67 kDa Laminin Receptor

Screening monoclonal antibodies for the ability to block infection, Wang et al. identified the 67 kDa laminin receptor protein (LAMR) as a receptor for Sindbis virus [60]. The presence and conservation of LAMR among distantly related species correlates with the broad host range of Sindbis virus. The transduction of LAMR cDNA into BHK cells was also found to increase levels of Sindbis virus infection. The study of LAMR was gaining interest at this time as earlier reports indicated its overexpression in some tumor cells [60].

LAMR has a high affinity for laminin, a major component of cell basement membranes that plays an important role in cellular adhesion, morphology, differentiation, and migration (reviewed in [61]). LAMR is a multifaceted protein that evolved from a 37 kDa ribosomal protein, although the nature of the precursor’s conversion from 37 to 67 kDa is still not well understood. It also has diverse functions and localizations within the nucleus, the ribosome, and the cell membrane (reviewed in [62]).

Many immunohistochemical studies of patient tumor biopsies went on to show increased levels of expression of LAMR on breast cancers [63,64,65], colon carcinomas [66,67], gastric cancer [68], lung cancer [69], pancreatic tumors [70], uterine adenocarcinoma [71], ovarian cancer [72,73,74,75], and thyroid cancer [76]. High levels of LAMR were shown in hepatocellular carcinoma via RT-PCR [77].

Recognizing the potential tumor targeting of SV vector via increased levels of LAMR on tumor cells motivated further studies to understand the association among SV, LAMR, and tumor cells. First, the replicon and envelope sequences of wild-type and laboratory-adapted SV strains were compared with respect to tumor targeting and suppression [53]. It was found that the SV Ar-339 wild-type strain provided the best suppression of tumor growth, whereas the E2 region of the Toto1101 Ar-339 showed better tumor targeting. A change of only one amino acid from E70 to K70 in the Ar-339 E2 protein suppressed the ability to target metastatic tumor implants in mice [53]. Further experiments utilized an SV vector derived from an AR-339 laboratory-adapted strain that contained amino acid E70 in the E2 envelope protein.

**Figure 3:**
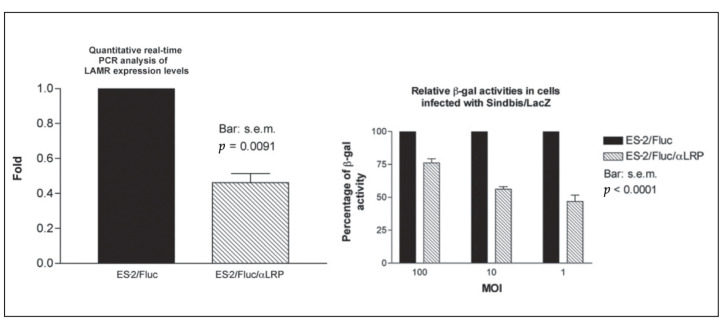
Infectivity of Sindbis vectors correlates with expression of the laminin receptor. ES-2/Fluc/αLRP are cells infected with pcDNA-Fluc/iLAMR.

To investigate the correlation between laminin receptor expression and Sindbis vector infection, a small interfering RNA (siRNA) was incorporated into a pcDNA-Fluc vector (pcDNA-Fluc/iLAMR) and stably transfected into ES-2 ovarian carcinoma cells. A quantitative RT-PCR indicated that the ES-2/Fluc/iLAMR cells expressed 40% of 37/67 kDa LAMR RNA levels compared with E2/Fluc cells (Figure 3, left panel). As hypothesized, the ES-2/Fluc/iLAMR cells expressing fewer laminin receptors were infected less effec tively by the Sindbis vectors compared with the ES-2/Fluc cells (Figure 3 right panel) [5].

**Figure 4:**
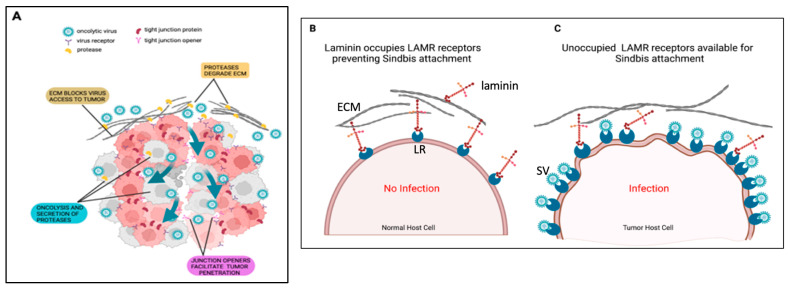
(**A**) Schematic of the tumor extracellular matrix being degraded, creating space for virus entry. (**B**) LAMRs in normal cells are bound to laminin and generally not overexpressed. (**C**) As a result of extracellular matrix degradation by metalloproteinases during metastasis, laminin detaches from LAMR, creating unoccupied receptors available for SV attachment. Additionally, LAMRs are overexpressed in tumors because of increased ribosome production, which increases targets for SV attachment and entry (via receptor-mediated endocytosis). ECM, extracellular matrix; LR, laminin receptor; SV, Sindbis virus vector. Created using BioRender.com.

Binding studies conducted with LAMR monoclonal antibodies suggest that tumor cells have varying degrees of unoccupied cell-surface LAMRs that conceivably results from the increased biosynthesis of LAMR or the dissolution of the surrounding basement membrane and extracellular matrix by tumor cells [78,79,80,81]. Thus, it is plausible that SV uses unoccupied LAMRs as fortuitous binding sites on tumor cells (Figure 4).

### 2.5. LAMR as a Cancer Therapy Target

The increased expression of LAMR on tumor cells and its interaction with the extracellular membrane (ECM) suggested a role for LAMR in tumor migration and metastases [61,80]. Blocking the LAMR–laminin interaction using antibodies, siRNA, or glycan molecules demonstrated a reduction in the invasiveness of a tumorigenic HT1080 fibrosarcoma cell line [82]. LAMR was also shown to modify laminin within the ECM to alter cell signaling [83] and tumor aggressiveness [84].

The connection between SV, LAMR, and tumorigenesis is intriguing. To understand LAMR functions an siRNA pool that targets different regions of LAMR and a non-targeting control siRNA was introduced into human cell lines. With this system, >90% of the LAMR RNA and protein were knocked down [85]. HeLa, HEK 293T, and HepG2 human cell lines transfected with the siLAMR were found to undergo cell-cycle arrest in the G1 phase, and the expression of cell-cycle-related RNA and protein were altered. At 37 kDa, LAMR is a ribosomal protein, and the effect of siLAMR on protein translation was examined. A striking reduction in protein synthesis was observed, along with a disruption of the poly-ribosomal profile.

**Figure 5:**
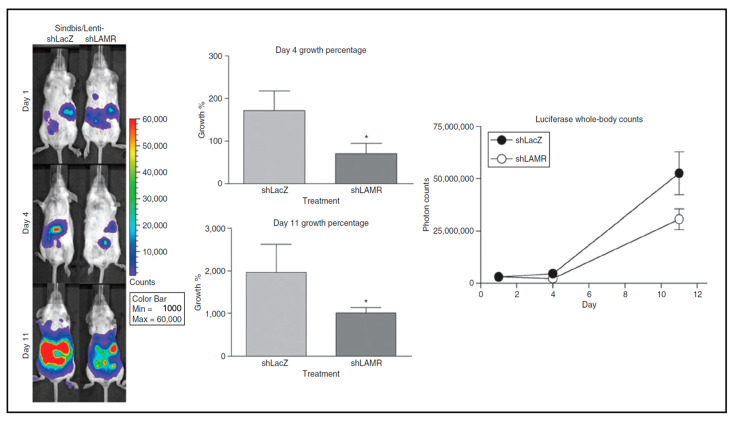
Targeting laminin receptor (LAMR) with Sindbis/Lenti pseudotype vectors inhibits ES-2 tumor growth in vivo. ES-2 cells express Fluc. IVIS RLU used to quantify tumor growth, color bar shows photo counts [85]. * *p* < 0.05, Student's *t*-test. [85].

To explore the effect of LAMR reduction in tumor cells, a Sindbis/Lentivirus pseudotype vector was developed which carried short-hairpin RNA (shRNA) designed against LAMR [85]. SCID mice with tumors from ES-2/FLuc cells were treated with the Sinbis/Lenti viruses (Figure 5). The mice that received treatment with the pseudotype vector that targets LAMR showed a reduction in tumor growth between ~60 and 50% tumor growth compared with a control pseudotype vector expressing LacZ on days 4 and 11, respectively. Further studies to understand how LAMR regulates cell survival revealed that the C-terminal 75 amino acid residues of LAMR were required for cell viability [86].

The function of LAMR as a cell-surface protein that interacts with the ECM and effects cell signaling and migration [83] prompted a study into the association of LAMR 37/67 kDa with the cytoskeleton. Other studies have shown that LAMR could alter gene expression [87] and cell degranulation [88] through its interactions with the cytoskeleton.

LAMR and actin play important roles in cell migration. When cells are plated on laminin, actin filaments are reorganized and lamellipodia are formed. LAMR is also required for components of the 40S ribosomal subunit to colocalize with α-tubulin and is important for protein translation [89]. A mutational analysis indicated that the binding sites for actin and tubulin are separate from the laminin binding site [90].

Several investigations have focused on 37/67 kDa LAMR as a target for tumor treatment. The knock-down of LAMR with siRNA blocked metastasis and induced apoptosis in MDA_MB231 human breast cancer cells and also inhibited telomerase activity [91]. Hypoxia-inducible factor-1 (Hif-1), which was found to increase 37/67 kDa LAMR and the metastases of human gastric tumor cells, showed diminished invasiveness when siRNA for either Hif-1 or LAMR was used in knock-down studies [92]. LAMR siRNA was also found to induce apoptosis in breast and esophageal cancer cells [93]. The application of a specific antibody for LAMR (IgG1-iS8) to melanoma cells inhibited invasion and adhesion [94]. The same antibody showed similar results for pancreatic cancer and neuroblastoma cells [95].

A LAMR tumor-associated antigen, designated oncofetal antigen immature laminin receptor protein (OFA-iLRP), was found to be immunogenic in rodent and human tumors (reviewed in [96]). OFA-iLRP was shown to be identical to the 37 kDa LAMR [97]. While the mature, dimeric, and modified 67 kDa LAMR becomes non-immunogenic, the 37 kDa OFA-iLRP remains immunogenic and is only expressed in early embryonic stages and is re-expressed in many types of cancer. Tumor vaccine studies conducted to induce antibodies in OFA-iLRP have led to early-phase clinical trials to treat renal cell carcinoma [98], lymphocytic leukemia [99], and hematological cancers [100]; however, no results have been published yet.

### 2.6. LAMR as a Receptor for Other Viruses

LAMR has been identified as a receptor for the alphavirus Venezuelan equine encephalitis virus (VEE) [101], several flavivirus serotypes, dengue [102,103], classical swine fever virus [104], West Nile virus [105], and the oncolytic adeno-associated DNA virus (AAV) [106]. Of these viruses, vectors generated from VEE have been tested in preclinical studies for human papilloma virus [107] and in clinical trials for prostate [108], breast [109], and metastatic cancers expressing a carcinoembryonic antigen (CEA) [110]. Adeno-associated virus vectors have also been used in several clinical trials (reviewed in [111]). In all these studies, however, levels of LAMR expression on tumor cells were not correlated with vector efficacy.

### 2.7. Additional Alphavirus Receptors

Although LAMR has been identified as a receptor for several viruses, it is important to recognize that the mechanisms of LAMR-mediated binding and cell internalization have not been determined. There are also instances in which cancer cells showing marked expression of LAMR were not infected very well [57,112]. Attachment factors that concentrate viruses at the cell surface, like heparan sulfate, C-type lectins, or phosphatidyl-serine residues, can also play a role in viral entry (reviewed in [11,113]). The possibility that defects in anti-viral IFN signaling in tumor cells affect tumor tropism has also been discussed [55,114].

Additional potential receptors were found more recently. A genome-wide RNAi screen in Drosophila cells revealed the natural resistance-associated macrophage protein (NRAMP), a divalent metal transport protein, as a receptor for Sindbis virus [115]. The vertebrate homolog NRAMP2 was shown to mediate the infection of mammalian cells with Sindbis; mouse cells rendered deficient for NRAMP2 were not infected with Sindbis. The association between Sindbis tumor targeting and NRAMP2 expression, however, has not been reported.

Affinity purification followed by mass spectrometry identified the CD147 membrane protein as a receptor for CHIKV, RRV, Eastern and Western equine encephalitis, and SV in human cells [116]. CD147 has been found to be overexpressed on many cancer cells and cells within the tumor microenvironment, and it plays a role in tumor proliferation and the inhibition of apoptosis [117].

Additional receptors have been detected via CRISPER/Cas9 screens for single-guide RNAs that prevent virus infection. The low-density lipoprotein receptor class A domain containing protein 3 (LDLRAD3) was found to be a VEE receptor [118]. VLDLR and ApoER2, members of the low-density lipoprotein family, were found to be receptors for SV, SFV, and Eastern equine encephalitis virus [119]. A matrix-remodeling-associated protein 8 (MXRA8) was shown to serve as a receptor for O’nyong nyong virus (ONNV), Mayaro virus (MAYV), CHIKV, SFV, and RRV [120]. Although it is not clear whether these receptors can be utilized as targets for cancer therapy, MXRA8 has been shown to be increased in thyroid carcinoma cells [121], is abundant in most solid tumors [122], and correlates with tumor cell aggressiveness and an immunosuppressive environment [123].

## 3. The Delivery of Anti-Tumor Agents

Extensive summaries of preclinical and clinical trials using alphavirus vectors to deliver anti-tumor agents for cancer therapy have been published [124,125,126]. Studies with SV, SFV, and VEE virus vectors are presented here.

### 3.1. Cytokines

Early studies indicated that the SV vector expression of IL-12 [6] and IL-15 [1] enhanced anti-tumor activity. Sin/IL12 was used to treat intraperitoneal tumors of ES-2 human ovarian carcinoma in SCID mice [35]. The efficacy of mouse survival was linked to natural killer cell (NK) activation. Treatment showed a significant intraperitoneal influx of NK cells and increased IFNγ levels that upregulated MHC class II expression on peritoneal macrophages. The effect of SV.IL12 was also tested in immunocompetent mice bearing CT26 colon cell tumors [4]. Although SV.IL12 produced a modest increase in survival, an examination of T cells one week after treatment showed the upregulation of OX40 on effector CD4 T cells and the stimulation of macrophages. This observation suggests the use of combined treatment (see Section 4.3). Notably, plasma IL-12 levels did not significantly increase with treatment, indicating that IL-12, produced at high levels in infected cells in vitro, was produced locally by the SV.IL12 vector in vivo and was, therefore, less likely to cause toxicity.

Semliki forest alphavirus (SFV) expressing IL-12 has been used in several pre-clinical trials. The co-administration of SFV-IL-12 and SFV-E6,E7 (oncogenic proteins of human papilloma virus) was shown to augment the induction of cytotoxic T cells, and 28% of mice remained tumor-free for 45 days [127]. The delivery of IL-12 via a self-amplifying RNA, derived from SFV intratumorally electroporated into mice, to treat MC38 subcutaneous colon and hepatocarcinoma tumors showed anti-tumor effects and the induction of CD8 T cells [128]. The intratumoral injection of an SFV-IL-12 vector resulted in the regression of a B16 murine melanoma. In this study, IFNγ was induced; however, no increases in NK cells or T-cell mediated cytotoxicity were observed. The tumor inhibition was attributed to the antiangiogenic activity of IL-12 [129]. Antiangiogenic activity and tumor suppression were also observed when SFV expressing murine vascular endothelial growth factor (VEGF2) was used to treat mice with CT26 colon carcinoma and 4T1 metastatic mammary carcinoma [130].

### 3.2. Tumor-Associated Antigens

Tumor-associated antigens (TAAs), predominantly expressed by tumor cells compared with normal cells, can be indirectly targeted by virus vectors encoding the TAA to stimulate an immune response. This process was demonstrated using a CT26 colon carcinoma cell line transduced to express β-galactosidase (LacZ), CT26.CL25 [31]. An SV vector encoding LacZ (SV/LacZ) was i.p. injected into immunocompetent mice bearing CT26.CL25 tumors. Although SV/LacZ does not infect CT26 tumors, a strong therapeutic effect was observed. Knowing that the intraperitoneal injection of SV/luciferase leads to an infection of the mediastinal lymph nodes (MLNs) within three hours, T cells within the MLN were analyzed 24 h after treatment. A significant influx of activated CD8 T cells was observed that were shown to be required for the antigen-specific effect of SV/LacZ. Surprisingly, splenocytes from the SV/LacZ-treated tumor-cured mice acquired cytotoxicity against not only CT26.CL25 cells but also LacZ-negative CT26.WT cells. An immune response to endogenous CT26.CL25 antigens was shown to occur via the process known as epitope spreading. This study is notable for the observation that the direct targeting of tumors is not necessarily required. The stimulation of a potent immune response via the SV vector delivery of antigens to lymph nodes can produce an effective tumor treatment.

Numerous studies of therapeutic TAAs expressed by alphaviruses were previously summarized [124,126,131]. An SFV vector encoding the P1A testis antigen, expressed on mast cell tumors, was shown to be effective at treating murine mastocytomas [124,126]. The SFV-P1A vector was shown to induce the highest level of tumor protection compared with adenovirus and vaccinia virus vectors expressing the P1A antigen [132].

Preclinical therapies for mouse melanoma used SV and VEE viruses to express the tyrosinase-related proteins (TRP), TRP-1 and TRP-2 and the adhesion proteins MCAM/MUC18. Mice immunized with a DNA Sindbis replicon vector encoding mouse TRP-1 was shown to provide significant protection against B16 melanoma [36]. An SV-based DNA plasmid was also used to express the melanoma antigen, MUC18. Vaccination with this vector reduced the incidence of B16 melanoma and metastasis and induced humoral and cellular immunity [133]. Similarly, a VEE-VRP expressing TRP-2 proved to be an effective therapeutic vaccine against melanoma in mice, providing long-term protection through the activation of antibodies and T cell responses [134].

Human epidermal growth factor (HER2)/neu proto-oncogene (neu) was expressed by a VEE-VRP to treat murine breast cancers [124,126,131]. VRP-neu vaccination prevented tumor formation and induced high levels of neu-specific CD8 T cells and anti-neu antibody [135].

To treat murine prostate tumors, VEE vectors producing prostrate membrane proteins, PMSA [136] and STEAP [137], were utilized.

Endostatin, an anti-angiogenic protein, was expressed from an SFV to treat brain tumors. This study highlighted the therapeutic importance of the anti-viral response to the alphavirus vector treatment as endostatin alone did not inhibit angiogenesis or tumor growth [138].

Human papilloma virus (HPV) oncogenic proteins E6 and E7 were expressed in SFV and VEE to treat murine cervical cancer [126,139]. A phase I trial of SFV-E6,E7 reported safety and immunological responses in 12/12 participants [140].

Vectors based on VEE with TAA for a carcinoembryonic protein were used in a phase I clinical trial for several metastatic tumors. The results were encouraging, showing improved survival and immune response. The prostate cancer PSMA antigen and breast cancer antigen HER2/neu expressed by VEE vectors have also entered phase I trials [125].

#### The Modification of TAAs

Several groups have modified TAAs to enhance their immunogenicity. Fusing the HPV E7 oncogene to HSP70 increased the efficacy of a Sindbis RNA replicon [141] and virus vector [142]. Calreticulin, which interacts with the transport proteins involved with antigen presentation, was also fused to the HPV E7 protein and expressed from an SV vector [143]. To compensate for the inability of the plasmid or virus-particle-based Sindbis vectors to proliferate, Cheng et al. fused HPV E7 to the Herpes Simplex virus VP22 protein, which possesses a transport function, to disperse E7 among tissue cells [144]. These strategies were found to increase T-cell-mediated responses and long-term tumor-specific immunity.

## 4. Combination Therapy with Immunomodulatory Proteins

### 4.1. SV-NYESO1 and PD-1 Antibody

**Figure 6:**
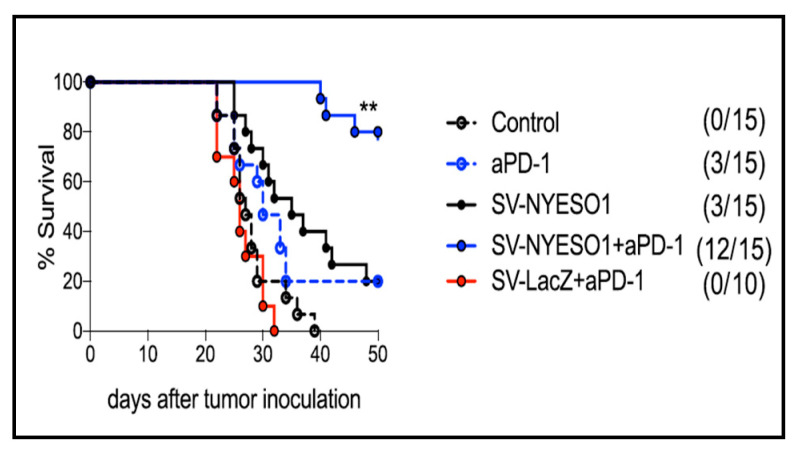
SV-NYESO1 plus anti-PD1 increases mouse survival. aPD-1, antibody to PD-1; **, significance determined by Mantel-Cox test *p* <0.01. [3].

Attempts to optimize the SV vector to achieve long-term curative results focused on combining SV vector therapy with immunomodulatory agents. CT26 colon carcinoma cells were transfected with the TAA NY-ESO-1, a human cancer testis antigen which is expressed in many tumoral cells but absent from normal tissue [145]. Tumors were established in syngenetic, immunocompetent BALB/c mice. In this model, the oncolytic effects of the SV vector treatment were precluded as CT26 cells are not susceptible to SV infection. As the CT26 cells were shown to express programmed death protein ligand 1 (PDL-1), a treatment combining SV-NYESO1 with the antibody to PD-1 was examined. Figure 6 shows that while the SV vector expressing either NY-ESO-1 or PD-1 antibody prolongs survival, the combination of SV-NYESO1 and the antibody to PD-1 resulted in significant tumor regression. Mice rechallenged with tumor cells after 200 days remained tumor-free. A further analysis of T cells from splenocytes and lymph nodes showed that SV-NYESO1 is a potent immunostimulatory agent that induced a systemic pro-inflammatory reaction and lymphocyte activation within 2 days. The addition of the anti-PD-1 checkpoint to SV-NYESO1 therapy induced a stronger systemic and an intratumoral immune response indicated via increased T cell cytotoxicity and the production of IFNγ [3]. Importantly the results showed that the SV infectivity of macrophage or antigen-presenting cells within lymph nodes was sufficient to induce a systemic immune response.

### 4.2. SV Vector and 4-1BB Agonistic Antibody

**Figure 7:**
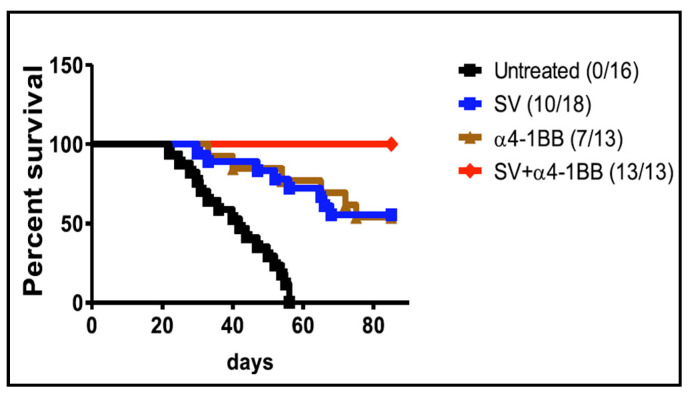
Survival curve of BALB/c mice with A20 lymphoma treated with SV vector and anti-4-1BB alone and in combination. Ratio is survived number/total number [147].

Following the observation that potent anti-lymphoma activity was elicited by an agonistic antibody to the T cell co-stimulatory molecule 4-1BB (CD137) [146], anti-4-1BB was combined with an empty SV vector to treat an A20 B cell lymphoma BALB/c tumor model [147]. All mice receiving combined treatment survived long-term (Figure 7). Mice surviving >4 months and rechallenged with A20 tumor cells all survived. Significantly, this study indicated that the expression of a TAA was not required for an SV vector plus anti-41BB to successfully treat A20 tumors.

To understand the nature of survival, at 7 days after treatment, T cells purified of splenocytes and tumor cells were analyzed via RNA-seq transcriptomics, ELISPOT, flow cytometry, and cytotoxicity and metabolic assays. The results indicated that combined treatment enhances T cell proliferation, activation, migration, and cytotoxicity. An influx of activated and metabolically energized T cells were found within the tumor mass, while immune-suppressive T reg cells were decreased [147].

### 4.3. SV.IL12 and Anti-OX40 Antibody

As mentioned in Section 3.1, an SV vector expressing IL-12 (SV.IL12) activates T cells and increases the expression of OX40 on CD4 cells. OX40 is a TNF-family co-stimulatory receptor that is expressed on activated T cells [148] and also represses regulatory T cell induction [149]. Activated OX40 promotes the function and survival of Th1, a proinflammatory T cell [150].

**Figure 8:**
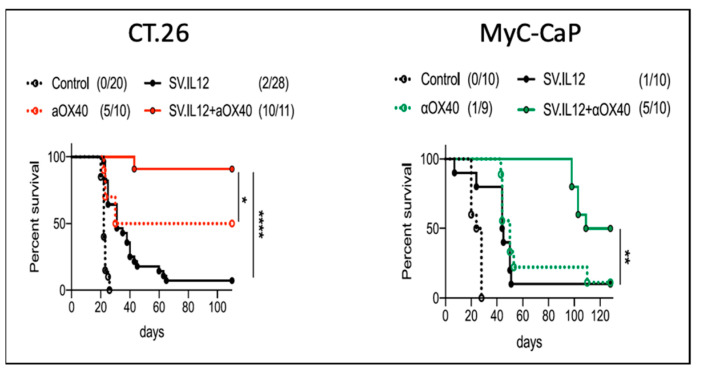
Survival curves of control and SV vector treated mice in two tumor models. Statistical analysis determined with Mantel-Cox test *, *p* < 0.05; **, *p* < 0.005; ****, *p* < 0.00005 [4].

To test the effect of SV.IL12 with the agonistic anti-OX40 antibody, SV-susceptible prostate cancer, MyC-CaP, and non-susceptible CT26 colon cancer tumor cell models were injected into immunocompetent FVB/NJ and BALB/c mice, respectively. As shown in Figure 8, the combined SV.IL12 and anti-OX40 (αOX40) treatment showed a significant regression of tumors in the two distinct models. Again, the oncolytic activity of SV was not required to stimulate an effective anti-tumor response.

**Figure 9:**
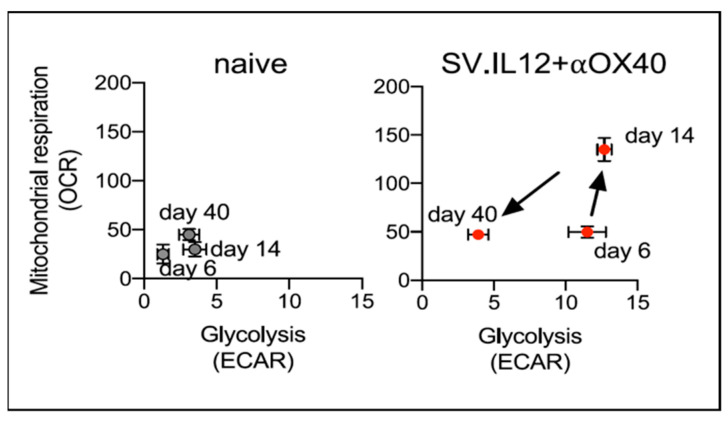
SV.IL12 plus anti-OX40 promotes metabolic programming of T cells.

Splenocytes and tumor cells were analyzed, as in Section 4.2. Collectively, the results demonstrated that only the combined treatment with SV.IL12 and αOX40 generated the induction of differentiated effector T cells. Metabolic assays of CT.26 splenic T cells indicated that the combined treatment boosted T cells to a highly energetic state by 14 days (Figure 9), a hallmark of highly activated T cells [151].

The combined treatment increased the infiltration of T cells into the tumor, as assessed via flow cytometry and multiplex immunofluorescence. Flow cytometry showed that the CD4 T cells expressed ICOS^+^T-bet+ and granzyme B, which are indicative of an anti-tumor phenotype. The CD4 T cells were also found to produce the highest levels of IFNγ [4]. This observation is noteworthy as the role of CD4 anti-tumor cytotoxic lymphocytes, independent of CD8 T cells, is increasingly being recognized [152]. Overall, the data suggest that independent of the tumor model, the distinct T cell response induced by SV.IL12 plus αOX40 stems from the interaction with the peripheral immune cells that are activated to infiltrate the tumor to eliminate the tumor cells.

### 4.4. SV Vector Expressing αOX40

The combination of SV.IL12 and αOX40 was used to treat a mouse ovarian surface epithelial cell (MOSEC.ID8) that was adapted to be more stable in vivo (MOSEC.p11). MOSEC.p11 expresses firefly luciferase and is susceptible to SV infection. Initial experiments showed an increased migration of immune cells into tumor tissue when mice were treated with either SV.IL12, αOX40, or SVIL.12 plus αOX40. In metabolic studies, however, only a combined treatment stimulated highly energized T cells [2].

**Figure 10:**
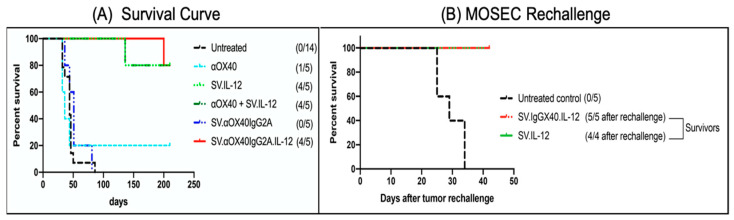
(**A**) Survival curve shows SV vector platform suppresses MOSEC ovarian tumors in vivo. (**B**) Survivors (140 days) rechallenged with MOSEC tumor cells are protected from tumor recurrence [2].

Studies in several mouse models indicate that the SV.IL12 vector in combination with the agonistic antibody for OX40 was efficacious and devoid of adverse effects. The encouraging results with the CT.26, MyC-CaP, and MOSEC.p11 tumor models motivated the generation of a single SV vector encoding both αOX40 and IL-12 to facilitate local delivery to peripheral lymphoid tissue and to tumors susceptible to SV infection [2]. This vector, SV.IgGOX40.IL12, effectively suppressed ovarian cancer tumors in vivo. Survivors rechallenged with MOSEC.p11 cells after 140 days were protected from tumor recurrence (Figure 10). In this ovarian cancer model, SV.IL12 was as effective as the combined therapy. As IL-12 stimulates OX40 expression on CD4 cells, it is likely that SV.IL12 tumor infection locally increases OX40 signaling.

Depletion studies indicated the importance of CD4 and CD8 T cells and, once again, CD4 T cells were shown to play a pivotal role in treatment effects. An RNA transcriptome analysis of tumor tissue also indicated that the treatment reprogrammed cells within the tumor to express genes related to an upregulated immune response.

### 4.5. Alphavirus Combined with Immunotherapy

Other alphaviruses have been used in conjunction with immunomodulatory agents. Co-immunizations with SFV vectors encoding VEGF-2 and IL-4 were found to induce high titers of VEGF antibodies and increase the survival of mice bearing CT.26 colon cancer or 4T1 metastatic breast cancer [130]. SFV-IL-12 and an anti-PD-1 antibody were able to synergistically treat B16 melanoma and MC38 colorectal tumors [153,154]. A VEE vector expressing a tyrosinase-related protein TAA , was used in combination with the antagonistic anti-CTLA-4 antibody or the agonistic GITR antibody to treat a B16F10 mouse melanoma model. The addition of the immunomodulatory antibodies increased the immunity of the VEE-TAA vector alone [155]. A phase II clinical trial testing a VEE-based vector, VRP-HER2, combined with an anti-PD-1 antibody produced promising results. Tumor biopsies, sera, and peripheral blood mononuclear cells (PBMCs) were collected before and after the immunizations. The vector treatment showed overall enhancement of the immune response with the combined treatment [125].

Table 1 and Table 2 present preclinical and clinical trials performed within the last 5 years using alphaviruses. In most cases, the alphavirus vectors combine the expression of additional agents with their oncolytic action. Previous preclinical and clinical trials with alphaviruses have been reported in recent reviews [124,125,156].

## 5. Summary

Sindbis virus has emerged as a viable vector for the treatment of cancer. Imaging studies have demonstrated a natural proclivity of Sindbis vectors for tumor cells, achieved through direct infection or indirectly through an interaction with peripheral lymphoid tissue, thereby augmenting and mobilizing pre-existing immune cells to target tumors. In several preclinical in vivo models, SV vectors have inhibited tumor growth and conferred long-term protection against tumor rechallenge. Treatment efficacy is dependent upon T-cells that are transcriptionally reprogrammed, leading to the expression of immune response genes and metabolic alterations that result in higher energy states. A notable influx of immune cells into the tumor microenvironment occurs.

A Sindbis virus (SV) vector platform has been developed, combining the expression of interleukin-12 (IL-12) and an agonistic antibody targeting the co-stimulatory OX40 receptor. This platform has yielded promising results in treating various preclinical mouse models. Its translation to clinical applications is feasible as SV vectors can be produced and purified under Good Manufacturing Practice (GMP) standards, ensuring high titers (10^11^ transducing units per milliliter (Tu/mL)) to compensate for dilution in the bloodstream.

As mentioned in Section 1.1 and Section 1.2, Sindbis virus vectors produce high levels of expressed proteins over a short period of time (24–48 h), building a robust T cell priming in the lymph nodes. Despite this immune activation, in some cases, treatment efficacy may require multiple doses. While alphaviruses are not highly immunogenic, the requirement for many doses could pose challenges and may lead to patient reluctance to undergo treatment. New vector designs to improve efficacy with fewer doses are now being studied in preclinical models. The lack of clinical trials involving Sindbis virus vectors thus far makes it difficult to evaluate their transition from animal models to human patients.

A number of other alphavirus vectors, primarily based on Venezuelan equine encephalitis (VEE) or Semliki Forest virus (SFV), have been generated for cancer treatment and cancer immunotherapies. Some vectors have advanced to clinical trials, demonstrating promising safety profiles and therapeutic potential. The continued optimization of vectors and administration protocols is expected to enhance the therapeutic efficacy of these versatile vectors.

## Figures and Tables

**Table 1:** **Preclinical Trials: Alphavirus Vectors.** αPD-1, programmed cell death check point antibody; VRP, vector replicative particle; i.p., intraperitoneal; i.m., intramuscular; i.t., intratumoral; i.v., intravenous; TILs, tumor infiltrating lymphocytes; Ki-67, proliferation marker; ND, not determined; M1, alphavirus that targets ZAP (-) tumor cells.

Vector/Agent	Delivery	Cancer	Immune Response	Clinical Outcome	
SV-NY-ESO-1 + α PD-1 (VPR)	i.p.	colon	T cell activation TILs	tumor growth inhibition	[3]
SV + α 4-1BB (VRP)	i.p.	B cell Lymphoma	T cell activation	tumor growth inhibition, protection against rechallenge	[147]
SV-IL12 (VPR) SV-IL12 + α OX40	i.p.	colon prostate	T cell activation, metabolic reprogramming TILs	tumor elimination	[4]
SV-IL12 + α OX40 (VPR)	i.p.	ovarian	T cell activation, metabolic reprogramming TILs	tumor elimination, protection against rechallenge	[2]
VEE-HPV-E6/E7 (VRP)	i.m.	cervical	CD8 T cells, IFNγ T memory cells	protection against rechallenge	[107]
SFV- α PDL-1 (VPR)	i.t.	colon	T cells, IFNγ,TILs	>40% tumor regression	[154]
M1	i.v.	bladder	Lower Ki-67 signals in tumor	tumor growth inhibition, increased survival	[157]
M1 + doxorubicin	i.v.	breast	ND	tumor growth inhibition	[158]

**Table 2:** **Clinical Trials: Alphavirus vectors.** (SV PV), Sindbis pseudovirus; i.d., intradermal; OS, overall survival; CEA, carcinoembryonic antigen; E6/E7, HPV.

Vector/Agent	Delivery	Cancer	Immune Response	Clinical Outcome	
Phase I VEE(VPR)-CEA	i.m.	Stage III colon	T cells, IFNγ αCEA	Improved OS in IFNγ (+) patients	[125]
Phase I Vvax001 SFV-E6/E7	i.m.	cervical	T cells, IFNγ Th1 response	ND	[159]
Phase I LV305 LV-NY-ESO-1 (SV PV)	i.d.	metastatic NY-ESO-1 (+) tumor	T cell response in 85% patients	tumor growth inhibition improved OS	[160]

## Data Availability

All sequencing data that support the findings of this study will be deposited in the National Center for Biotechnology Information Gene Expression Omnibus (GEO) and are accessible through the GEO Series accession number that will be provided, including all other relevant data included in the article. Further inquiries can be directed to the corresponding authors.
